# Supplementary material for: Heat adaptation of phage T7 under an extended genetic code
Source: Virus Evol. 2021 Dec 1;7(2):veab100. doi: 10.1093/ve/veab100 (PMC8923235; doi:10.1093/ve/veab100)
Supplement: veab100_Supp [file veab100_supp.zip › Sequencing_Analysis.zip!/Sequencing_Analysis/S1/evidence/SNP_6.html]

BRESEQ :: Evidence


| Predicted mutation | | | | | | |
| --- | --- | --- | --- | --- | --- | --- |
| evidence | seq id | position | mutation | annotation | gene | description |
| RA | NC\_001604.1 | 24,238 | A→G | intergenic (–/–) | – / – | –/– |

| Read alignment evidence... | | | | | | | | | | | |
| --- | --- | --- | --- | --- | --- | --- | --- | --- | --- | --- | --- |
|  | seq id | position | | ref | new | freq | score (cons/poly) | reads | annotation | genes | product |
| \* | NC\_001604.1 | 24,238 | 0 | A | G | 98.5% | 11802.7 / NA | 4006 | intergenic (–/–) | *–/–* | –/– |
| Reads supporting (aligned to +/- strand):  **ref** base A (10/4);  **major** base G (2033/1893);  **minor** base T (37/23);  **total** (2085/1921) | | | | | | | | | | | |
| Fisher's exact test for biased strand distribution *p*-value = 1.52e-01 | | | | | | | | | | | |
| Kolmogorov-Smirnov test that lower quality scores support variant *p*-value = 1.00e+00 | | | | | | | | | | | |

Only 100 of 4006 total aligned reads displayed.  

`GTACGCAATGGGCCACGGTGGTCTTCGCCCAGAAGCTGCTGGTGCAGTGGTTTTCAAAGTGGAGTAATGC‑TGGGGGTGGCCTCAACGGTCGCTGCTAGTCCCGAAGAGGCGAGTGTTACTTCAACAGAAGAAACCTTAACGCCAGCACAGGAGGCCGCACGCACCCGCGCTGCTAACAAAGCCCGAAAGGAAGCTGAGTTGGCTGCTGCCACCGCTGAGCAATAACTAGCATAACCCCTTGGGGCCTCTAAACGGGTCTTGAGGGGTTTTTTGCTGAAAGGAGGAACTATATGCGCTCATACGATATGAACGTTGAGACTGCCGCTGAGTTATCAGCTGTGAACGACATTCTGGCGTCTATCGGTGAACCTCCGGTATCAACGCTGGAAGGTGACGCTAACGCAGATGCAGCGAACGCTCGGCGTATTCTCAACAAGATTAACCGACAGATTCAATCTCGTGGATGGACGTTCAACATTGAGGAAGGCATAACGCTACTACCTGATGTTTACTCCAACCTGATTGTATACAGTGACGACTATTTATCCCTAATGTCTACTTCCGGTCAATCCATCTACGTTAACCGAGGTGGCTATGTG  >  NC_001604.1/23938‑24536`  

                                                                                                                                                                                                                                                                                                             `|`                                                                                                                                                                                                                                                                                                            
            `ccACGGTGGTCTTCGCCCAGAAGCTGCTGGTGCAGTGGTTTTCAAAGTGGAGTAATGC‑TGTGGGTGGCCTCAACGGTCGCTGCTAGTCCCGAAGAGGCGAGTGTTACTTCAACAGAAGAAACCTTAACGCCAGCACAGGAGGCCGCACGCACCCGCGCTGCTAACAAAGCCCGAAAGGAAGCTGAGTTGGCTGCTGCCACCGCTGAGCAATAACTAGCATAACCCCTTGGGGCCTCTAAACGGGTCTCGAGGGGTTTTTTGCTGAAAGGAGGAACTATATGCGCTCATGCGATATGAACGt                                                                                                                                                                                                                                                                                                >  1:209826/1‑301 (MQ=255)`  
              `aCGGTGGTCTTCGCCCAGAAGCTGCTGGTGCAGTGGTTTTCAACGTGGATTAATGC‑TGGGGGAGGCCTCAACGGTCGCTGCTAGTCCCGAAGAGGCGAGTGTTACTTCAACAGAAGAAACCTTAACGCCAGCACAGCAGGCCGTACGCCCCCGCGCTGCTAACAAACCCCGAAAGGAAGCTGAGGCACCTGCTGCCACCGCGAAGCAAAAACTAGAATAACCCCTTGGTGCCTCTAAACGGGTATTGAGGGGTGTTTTGCTGACAGGGGGAACTAAATGCGCTCATGCGAGATGatagtgg                                                                                                                                                                                                                                                                                              >  1:631324/1‑295 (MQ=255)`  
               `cGGTGGTCTTCGCCCAGAAGCTGCTGGTGCAGTGGTTTTCAAAGTGGAGTAATGC‑TGGGGGTGGCCTCAACGGTCGCTGCTAGTCCCGAAGAGGCGAGTGTTACTTCAACAGAAGAAACCTTAACGCCAGCACAGGAGGCCGCACGCACCCGCGCTGCTAACAAAGCCCGAAAGGAAGCTGAGTTGGCTGCTGCCACCGCTGAGCAATAACTAGCATAACCCCTTGGGGCCTCTAAACGGGTCTTGAGGGGTTTTTTGCTGAAAGGAGGAACTATATGCGCTCATGCGATATGAACGTTgc                                                                                                                                                                                                                                                                                             >  1:128648/1‑300 (MQ=255)`  
                `ggtggtCTTCGCCCAGAAGCTGCTGGTGCAGTGGTGTTCAAAGTGGAGTAATGC‑TGGGGGTGGCCTCAACGGTCGCTGCTAGTCCCGAAGAGGCGAGTGTTACTTCAACAGAAGAAACCTTAACGCCAGCACAGGATGCCGCACGCACCCGCGCTGCTAACAAAGCCCGAAAGGAAGCTGAGTTGGCTGCTGCCACCGCTGAGCAATAACTAGCATAACCCCTTGGGGCCTCTAAACGGGTCTTGAGGGGTTTTTTGCTGAAAGGAGGAACTATATGCGCTCATGCGATATGAACGTTgag                                                                                                                                                                                                                                                                                            <  1:100003/301‑1 (MQ=255)`  
                `aaagagacaggCCCAGAAGCTGCTGGTGCAGTGGTTTTCAAAGTGGAGTAATGC‑TGGGGGTGGCCTCAACGGTCGCTGCTAGTCCCGAAGAGGCGAGTGTTACTTCAACAGAAGAAACCTTAACGCCAGCACAGGAGGCCGCACGCACCCGCGCTGCTAACAAAGCCCGAAAGGAAGCTGAGTTGGCTGCTGCCACCGCTGAGCAATAACTAGCATAACCCCTTGGGGCCTCTAAACGGGTCTTGAGGGGTTTTTTGCTGAAAGGAGGAACTATATGCGCTCATGCGATATGAACGTTgag                                                                                                                                                                                                                                                                                            <  1:266398/291‑1 (MQ=255)`  
                    `gtCTTCGCCCAGAAGCTGCTGGTGCAGTGGTTTTCAAAGTGGAGTAATGC‑TGGGGGTGGCCTCAACGGTCGCTGCTAGTCCCGAAGAGGCGAGTGTTACTTCAACAGAAGAAACCTTAACGCCAGCACAGGAGGCCGCACGCACCCGCGCTGCTAACAAAGCCCGAAAGGAAGCTGAGTTGGCTGCTGCCACCGCTGAGCAATAACTAGCATAACCCCTTGGGGCCTCTAAACGGGTCTTGAGGGGTTTTTTGCTGAAAGGAGGAACTATATGCGCTCATGCGATATGAACGTTGAGACTg                                                                                                                                                                                                                                                                                        >  1:614103/1‑301 (MQ=255)`  
                                  `gctgctGGTGCAGTGGTTTTCAAAGTGGAGTAATGCGGGGGGGGGGCCTCAACGGTCGCTGCTAGTCCCGAAGAGGCGAGTGTTACTTCAACAGAAGAAACCTTAACGCCAGCACAGGAGGCCGCACGCACCCGCGCTGCTAACAAAGCACGAAAGGAAGCTGAGTTGGCTGCTGCCACCGCTGAGCAATAACTAGCATAACCCCTTGGGGCCTCTAAACGGGTCTTGAGGGGTTTTTTGCTGAAAGGAGGAACTATATGCGCTCATGCGactgtctcttatacacatctccgagcccacg                                                                                                                                                                                                                                                                           >  1:562941/1‑271 (MQ=255)`  
                                         `gTGCAGTGGTTTTCAAAGTGGAGTAATGC‑TGGGGGTGGCCTCAACGGTCGCTGCTAGTCCCGAAGAGGCGAGTGTTACTTCAACAGAAGAAACCTTAACGCCAGCACAGGAGGCCGCACGCACCCGCGCTGCTAACAAAGCCCGAAAGGAAGCTGAGTTGGCTGCTGCCACCGCTGAGCAATAACTAGCATAACCCCTTGGGGCCTCTAAACGGGTCTTGAGGGGTTTTTTGCTGAAAGGAGGAACTATATGCGCTCATGCGATATGAACGTTGAGACTGCCGCTGAGTTATCAGCTGTGa                                                                                                                                                                                                                                                                   >  1:81146/1‑301 (MQ=255)`  
                                           `gCAGTGGTTTTCAAAGTGGAGTAATGC‑TGGGGGTGGCCTCAACGGTCGCTGCTAGTCCCGAAGAGGCGAGTGTTACTTCAACAGAAGAAACCTTAACGCCAGCACAGGAGGCCGCACGCACCCGCGCTGCTAACAAAGCCCGAAAGTAAGCTGAGTTGGCTGCTGCCACCGATGAGCAATAACTAGCATAACCCCTTGGGGCCTCTAAACGGGTCTTGAGGGGTTTTTTGCTGAAAGGAGGAACTATATGCGCTCATGCGATATGAACGTTGAGACTGCCGCTGAGTTATCAGCtgtctct                                                                                                                                                                                                                                                                 >  1:493759/1‑297 (MQ=255)`  
                                                     `tgtataagagaaagtGC‑TGGGGGGGGCCTCAACGGGCGCTGCTAGGCCCGAAGAGGCGAGTGTTACTTCAACAGAAGAAACCTTAACGCCAGCACAGGAGGCCGCACGCACCCGCGCTGCTAACAAAGCCCGAAAGGAAGCTGAGTTGGCTGCTGCCACCGCTGAGCAATAACTAGCATAACCCCTTGGGGCCTCTAAACGGGTCTTGAGGGGTTTTTTGCTGAAAGGAGGAACTATATGCGCTCATGCGATATGAACGTTGAGACTGCCGCTGAGTTATCAGCTGTGAACGACATTCTgg                                                                                                                                                                                                                                                       <  1:29669/287‑1 (MQ=255)`  
                                                     `agagacaggAGTAATGC‑TGGGGGTGGCCTCAACGGTCGCTGCTAGTCCCGAAGAGGCGAGTGTTACTTCAACAGAAGAAACCTTAACGCCAGCACAGGAGGCCGCACGCACCCGCGCTGCTAACAAAGCCCGAAAGGAAGCTGAGTTGGCTGCTGCCACCGCTGAGCAATAACTAGCATAACCCCTTGGGGCCTCTAAACGGGTCTTGAGGGGTTTTTTGCTGAAAGGAGGAACTATATGCGCTCATGCGATATGAACGTTGAGACTGCCGCTGAGTTATCAGCTGTGAACGACATTCTgg                                                                                                                                                                                                                                                       <  1:473572/294‑1 (MQ=255)`  
                                                            `ggggTAATGC‑TGGTGGTGGCCTCCACGGTCGCGGCTAGTCCCGAAGAGGCTAGTGTTACTTCAACAGAACAAACCTTATCGCCCGCACAGGAGGCCGCACGCACCCTCGCTGCCAACAAAGCCCGAAAGGAAGCTGAGTTGGCTGCTGCCACCGCTGAGCAATAACTAGCATAACCCCTTGGGGCCTCTAAACGGGTCTTGAGGGGTTTTTTGCTGAAAGGAGGAACTATATGCGCTCATGCGATATGAACGTTGAGACTGCCGCTGAGTTATCAGCTGTGAACGACATTCTGGCGTCTAt                                                                                                                                                                                                                                                <  1:514700/298‑1 (MQ=255)`  
                                                            `ggAGTAATGC‑TGGGGGTGGCCTCAACGGTCGCTGCTAGTCCCGAAGAGGCGAGTGTTACTTCAACAGAAGAAACCTTAACGCCAGCACAGGAGGCCGCACGCACCCGCGCTGCTAACAAAGCCCGAAAGGAAGATGAGTTGGCTGCTGCCACCGCTGAGCAATAACTAGCATAACCCCTTGGGGCCTCTAAACGGGTCTTGAGGGGTTTTTTGCAGAAAGGAGGAACTAAAGGCTGCCATGCGATATGAAGGTTGAGACTGACGGGAGGGTATCAGCTGTGAACGAAATTCTGGAGTCtca                                                                                                                                                                                                                                                >  1:596094/1‑299 (MQ=255)`  
                                                              `aGTAATGC‑TGGGGGTGGCCTCAAAGGTCGCTGGTAGTCACGAAGAGGCGAGTGTTACTTCAACAGAAGAAACCTTAACGCCAGCACAGGAGGCCGCACGCACCCGCGCTGCTAACAAAGCCCGAAAGGAAGCTGAGTTGGCTGCTGCCACCGCTGAGCAATAACTAGCATAACCCCTTTGGGCCTCTAAACGGGTCTTGAGGGGTTTTTTGCTGAAAGGAGGAACTATATGCGCTCATGCGATATGAACGTTGAGACTGCCGCTGAGTTATCAGCTGTGAACGACATTCTGGCGTCTATCg                                                                                                                                                                                                                                              <  1:216643/301‑1 (MQ=255)`  
                                                                `tgtataagagacagagCCTCAACGGTCGCTGATAGTCCCGAAGAGGCGAGTGTTACTTCAACAGAAGAAACCTTAACGCCAGCACAGGAGGCCGCACGCACCCGCGCTGCTAACAAAGCCCGAAAGGAAGCTGAGTTGGCTGCTGCAACCGCTGAGCAATAACTAGCATAACCCCTTGGGGCCTCTAAACGGGTCTTGAGGGGTTTTTTGCTGAAAGGAGGAACTATATGCGCTCATGCGATATGAACGTTGAGACTGCCGCTGAGTTATCAGCTGTGAACGACATTCTGGCGTCTATCgg                                                                                                                                                                                                                                             <  1:229402/286‑1 (MQ=255)`  
                                                                 `aaTGC‑GGGGGGTGGCCTCAACGGTCGCTGCTAGTACCGAAGAGGCGAGTGTTACTTCAACAGAAGAAACCTTAACGCCAGCACAGGAGGCCGCACGCACCCGCGCTGCTAACAAAGCCCGAAAGGAAGATGAGTTGGCTGCTGCCACCGCTGAGCAATAACTAGCATAACCCCTTGGGGCCTCTAAACGGGTCTTGAGGGGTTTTTTGCTGAAAGGAGGAAATATATGCGCTCATGCGATATGAACGTTGAGACTGCCGCTGAGTTATCAGCTGTGAACGACATTCTGGCGTCTATCGGTg                                                                                                                                                                                                                                           <  1:199550/301‑1 (MQ=255)`  
                                                                  `aTGC‑TGGGGGTGGCCTCAACGGTCGCTGCTAGTCCCGAAGAGGCGAGTGTTACTTCAACAGAAGAAACCTTAACGCCAGCACAGGAGGCCGCACGCACCCGCGCTGCTAACAAAGCCCGAAAGGAAGCTGAGTTGGCTGCTGCCACCGCTGAGCAATAACTAGCATAACCCCTTGGGGCCTCTAAACGGGTCTTGAGGGGTTTTTTGCTGAAAGGAGGAACTATATGCGCTCATGCGATATGAACGTTGAGACTGCCGCTGAGTTATCAGCTGTGAACGACATTcctgtctcttatacaca                                                                                                                                                                                                                                          >  1:236454/1‑285 (MQ=255)`  
                                                                        `gggggTGGCCTCAACGGTCGCTGCTAGTCCCGAAGAGGCGAGTGTTACTTAAACAGAAGAAACCTTAACGCCAGCACAGTAGGCCGCACGCACCCGCGCTGCTAACAAAGCCCGAAAGGAAGCTGAGTTGGCTGCTGCCACCGCTGAGCAATAACTAGCATAACCCCTTGGGGCCTCTAAACGGGTCTTGAGGGGTTTTTTGCTGAAAGGAGGAACTATATGCGCTCATGCGATATGAACGTTGAGACTGCCGCTGAGTTATCAGCTGTGAACGACATTCTGGCGTCTATCGGTGAACCTc                                                                                                                                                                                                                                     <  1:320222/301‑1 (MQ=255)`  
                                                                              `ggCCTCAACGGTCGCTGCTAGTCCCGAAGAGGCGAGTGTTACTTCAACAGAAGAAACCTTAACGCCAGCACAGGAGGCCGCACGCACTAGCGATGGTAACAAAGCACGAAAGGAAGCTGAGTTGGCTGATGCTACCGCTGAGCAATAACTAGCATAACCCCTTGGGGCCTCTAAACGGGTCTTGAGGGGCATATTGCTGAAAGGAGGAACTATATGCGCTCATGCGATATGAACGTCTATACTGCCGCTGAGTTTTAAGCTGTGAATGAAAATCTTGCATCTACCGGTGAACCTCCTGttt                                                                                                                                                                                                                               >  1:382181/1‑299 (MQ=255)`  
                                                                                         `tCGCTGCTAGGCCCGAAGAGGCGAGTGTTACTTCAACAGAAGAAACCTTAACGCAAGAACAGGAGGCCGCACGCACCCGCGCTGCTAACAAAGCCCGAAAGGAAGCTGAGTTGGCTGCTGACACCGCTGAGCAATAACTAGCATAACCCCTTGGGGCCTCTAAACGGGTCTTGAGGGGGTTTTTGCTGAAAGGAGGAAATATATGCGCTCATGCGATATGAACGTGGAGACTGCCGCTGAGTTATCAGCTGTGAACGACATTCTGGCGTCTATCGGTGAACCTCCGGTATCAACGCTGGaa                                                                                                                                                                                                                    <  1:204346/301‑1 (MQ=255)`  
                                                                                         `aagagccagggcccGAAGAGGCGAGTGTTACGTCAACAGAAGAAACCTTAACGCCAGCACAGGAGGCCGCACGCACCCGCGCTGCTAACAAAGCCCGAAAGGAAGCTGAGTTGGCTGCTGCCACCGCTGAGCAATAACTAGCATAACCACTTGGGGCCTCTAAACGGGTCTTGAGGGGTTTTTTGCTGAAAGGAGGAACTATATGCGCTCATGCGATATGAACGTTGAGACTGCCGCTGAGTTATCAGCTGTGAACGACATTCTGGCGTCTATCGGTGAACCTCCGGTATCAACGCTGGaa                                                                                                                                                                                                                    <  1:17762/290‑1 (MQ=255)`  
                                                                                                            `ggagAGTGTTACTTCAACAGAAGAAACCTTAACGCCAGCACAGGAGGCCGAACGCACACGAGCTGCTAACAAAGCCCGAAAGGAAGCTGAGTTGGCTGCTGCCACCGCTGAGCAATAACTAGCATAACCCCTTGGGGCCTCTAAACGGGTCTTGAGGGGATTTTTGCTGAAAGGAGGAACTATATGCGCTCATGCGATATGAACGTTGAGACTGCCGCTGAGTTATCAGCTGTGAACGACATTCTGGCGTCTATCGGTGAACCTCCGGTATACACGCTGGAAGGTGACGCTAACGCTGATg                                                                                                                                                                                                 >  1:308065/4‑301 (MQ=255)`  
                                                                                                                 `gtgtTACTTCAACAGAAGAAACCTTAACGCCAGCACAGGAGGCCGCACGCACCCGCGCTGCTAACAAAGCCCGAAAGGAAGCTGAGTTGGCTGCTGCCACCGCTGAGCAATAACTAGCATAACCCCTTGGGGCCTCTAAACGGGTCTTGAGGGGTTTTTTGCTGAAAGGAGGAACTATATTCGCTCATGCGATATGAACGTTGAGACTGCCGCTGAGTTATCAGCTGTGAACGACATTCTGGCGTCTATCGGTGAACCTCCGGTATCAACGCTGGAAGGTGACGCTAACGCAGATGCAGCg                                                                                                                                                                                            >  1:35345/1‑301 (MQ=255)`  
                                                                                                                 `gtgtTACTTCAACAGAAGAAACCTTAACGCCAGCACAGGAGGCCGCACGCACCCGCGCTGCTAACAAAGCCCGAAAGGAAGCTGAGTTGGCTGCTGCCACCGCTGAGCAATAACTAGCATAACCCCTTGGGGCCTCTAAACGGGTCTTGAGGGGGTTTTTGCTGAAAGGAGGAACTATATGCGCTCATGCGATATGAACGTTGAGACTGCCGCTGAGTTATCAGATGGGAACGACATTGTGGAGGCTATCGGTGAAGCTCCGGTAGCAACGCTGGAAGGTGACGCTAACGAAGATGCAGCg                                                                                                                                                                                            >  1:38713/1‑301 (MQ=255)`  
                                                                                                                              `agaagaAACCTTAACGCCAGCACAGGAGGCCGCACGCACCCGCGCGGCTAACAAAGCCCGAAAGGAAGCTGAGTTGGCTGCTGCCACCGCTGAGCAATAACTAGCATAACCCCTTTGGGCCTCTAAACGGGTCTTGAGGGGTTTTTTGCTGAAAGGAGGAACTATATGCGCTCATGCGATATGAACGTTGAGACTGCCGCTGAGTTATCAGCTGTGAACGACATTCTGGCGTCTATCGGTGAACCTCCGGTATCAACGCTGGAAGGTGACGCTAACGCAGATGCAGCGAACGCTCGACGTa                                                                                                                                                                               <  1:443060/301‑1 (MQ=255)`  
                                                                                                                                `agaaaaCCTGAACGCC‑GACCAGGAGGCCGCCAGCAACCGGGCTTGTAAAAAAGGCCGAAAGGAAGGGGAGGTGGCTGCTGGAAACGGTTGGTAATAAAGAGGAAAAACCCCTGGGGCCTTTAAACCGGGTGTGAGGGGGTTTTTGCGGAAAGGAGGAAATATATTGGGTCAAGGGATATGAACGTTGGGAAGGGCGAAGAGTTATCAGCTGTGAACGACATTCTGGCGTCTATCGGTGAACCTCCGGTATCAACGCTGGAAGGTGACGCTAACGCAGATGCAGCGAACGCTCGGCGTATtc                                                                                                                                                                            <  1:365434/298‑1 (MQ=255)`  
                                                                                                                                 `agaaACCTTAACGCCAGCACAGGAGGCCGGACGCACCCGCGCTGCTAACAAAGCCCGAAAGGAAGCTGAGTTGGCTGCTGCCACCGCTGAGCAATAACTAGCATAACCCCTTGGGGCCTCTAAACGGGTCTTGAGGGGTTTTTTGCTGAAAGGAGGAACTATATGCGCTCATGCGATATGAACGTTGAGACTGCCGCTGAGTTATCAGCTGTGAACGACATTCTGGCGTCTATCGGTGAACCTCCGGTATCAACGCTGGAAGGTGACGCTAACGCAGATGCAGCGAACGCTCGGCGTATtc                                                                                                                                                                            <  1:550645/301‑1 (MQ=255)`  
                                                                                                                                      `gatgtgtataagagacagaggCCGCACGCACCCGCGCTGCTAACAAAGCCCGAAAGGAAGCTGAGTTGGCTGCTGCCACCGCTGAGCAATAACTAGCATAACCCCTTGGGGCCTCTAAACGGGTCTTGAGGGGTTTTTTGCTGAAAGGAGGAACTATATGCGCTCATGCGATATGAACGTTGAGACTGCCGCTGAGTTATCAGCTGTGAACGACATTCTGGCGTCTATCGGTTAACCTCCGGTATCAACGCTGGAAGGTGACGCTAACGCAGATGCAGCGAACGCTCGGCGTATTCTcaac                                                                                                                                                                       <  1:117674/284‑1 (MQ=255)`  
                                                                                                                                      `ccTTAACGCCAGCACAGGAGGCCGCACGCACCCGCGCTGCTAACAAAGCCCGAAAGGAAGCTGAGTTGGCTGCTGCCACCGCTGAGCAATAACTAGCATAACCCCTTGGGGCCTCTAAACGGGTCTTGAGGGGTTTTTTGCTGAAAGGAGGCACTATATGCGCTCATGCGATATGAACGTTGAGACTGCCGCTGAGTTATCAGCTGTGAACGACATTCTGGCGTCTATCGGTGAACCTCCGGTATCAACGCTGGAAGGTGACGCTAACGCAGATGCAGCGAACGATCGGCGTATTCTcaaa                                                                                                                                                                       >  1:584673/1‑300 (MQ=255)`  
                                                                                                                                      `ccTTAACGCCAGCACAGGAGGCCGCACGCACCCGCGCTGCTAACAAAGCCCGAAAGGAAGCTGAGTTGGCTGCTGCCACCGCTGAGCAATAACTAGCATAACCCCTTGGGGCCTCTAAACGGGTCTTGAGGGGTTTTTTGCTGAAAGGAGGAACTATATGCGCTCATGCGATATGAACGTTGAGACTGCCGCTGAGTTATCAGCTGTGAACGACATTCTGGCGTCTATCGGTGAACCTCCGGTATCAACGCTGGAAGGTGACGCTAACGCAGATGCAGCGAACGCTCGGCGTATTCTcaac                                                                                                                                                                       >  1:339833/1‑301 (MQ=255)`  
                                                                                                                                           `aCGCCAGCACAGGAGGCCGCACGCACCCGCGCTGCTAACAAAGCCCGAAAGGAAGCTGAGTTGGCTGCTGCCACCGCTGAGCAATAACTAGCATAACCCCTTGGGGCCTCTAAACGGGTCTTGAGGGGTTTTTTGCTGAAAGGAGGAACTATATGCGCTCATGCGATATGAACGTTGAGACTGCCGCTGAGTTATCAGCTGTGAACGACATTCTGGCGTCTATCGGTGAACCTCCGGTATCAACGCTGGAAGGTGACGCTAACGCAGATGCAGCGAACGCTCGGCGTATTCTCAACAAGAt                                                                                                                                                                  <  1:479618/301‑1 (MQ=255)`  
                                                                                                                                              `ccAGCACAGGAGGCCGCACGCACCCGCGCTGCTAACAAAGCCCGAAAGGAAGCTGAGTTGGCTGCTGCCACCGCTGAGCAATAACTAGCATAACCCCTTGGGGCCTCTAAACGGGTCTTGAGGGGTTTTTTGCTGAAAGGAGGAACTATATGCGCTCATGCGATATGAACGTTGAGACTGCCGCTGAGTTATCAGCTGTGACCGACATTCTGGCGTCTATCGGTGAACCTCCGGTATCAACGCTGGAAGGTGACGCTAACGCAGAAGCAGCGAACGCACGGCGTATTCTCAACAAGATTaa                                                                                                                                                               >  1:501872/1‑301 (MQ=255)`  
                                                                                                                                               `cAGCACAGGAGGCCGCACGCACCCGCGCTGCTAACAAAGCTCGAAAGGAAGCTGAGTTGGCTGCTGCCACCGCTGAGCAATAACTAGCATAACCCCTTGGGGCCTCTAAACGGGTATTGAGGGGTTTTTTGCTGAAAGGAGGAAATATATGCGCTCATGCGATATGAACGTTGAGACTGCCGCTGAGTTATCAGCTGTGAACGACATTCTGGCGTCTATCGGTGAACCGCCGGTATCAACGCTGGAAGGTGACGCTAACGCAGATGCAGCGAACGCTCGGCGTATTCTCAACAAGATTAAc                                                                                                                                                              <  1:58044/301‑1 (MQ=255)`  
                                                                                                                                                `ggCACAGGAGGCCGCACGCACCCGCGCTGCTAACAAAGCCCGAAAGGAAGCTGAGTTGGCTGCTGCCACCGCTGAGCAATAACTAGCATAACCCCTTGGGGCCTCTAAACGGGTCTTGAGGGGTTTTTTGCTGAAAGGAGGAACTATATGCGCTCATGCGATATGAACGTTGAGACTGCCGCTGAGTTATCAGCTGTGAACGACATTCTGGCGTCTATCGGTGAACCTCCGGTATCAACGCTGGAAGGTGACGCTAACGCAGATGCAGCGAACGCTCGGCGTATTCTCAACAAGATTAAcc                                                                                                                                                             <  1:654664/300‑1 (MQ=255)`  
                                                                                                                                                 `gCACAGGAGGCCGCACGCACCCGCGCTGCTAACCAAGCCCGAAAGGAAGCTGAGTTGGCTGCTGCCACCGCTGAGCAATAACTAGCATAACCCCTTGGGGCCTCTAAACGGGTCTTGAGGGGTTTTTTGCTGAAAGGAGGAACTATATGCGCTCATGCGATATGAACGTTGAGACTGCCGCTGAGTTATCAGCTGTGAACGACATTCTGGCGTCTATCGGTGAACCTCCGGTATCAACGCTGGAAGGTGACGCTAACGCAGATGCAGCGAACGCTCGGCGTATTCTCAACAAGATTAACCg                                                                                                                                                            <  1:172354/301‑1 (MQ=255)`  
                                                                                                                                                       `gaggCCGCACGCACCCGCGCTGCTAATAAAGCCCGAAAGGAAGCTGAGTTGGCTGCTGCCACCGCTGAGAAATAACTAGCATAACCCCTTGGGGCCTCTAAACGGGTCTTGAGGGGTTTTTTGCTGAAAGGAGGAACTATATGCGCTCATGCGATATGAACGTTGAGACTGCCGCTGAGTTATCAGCTGTGAACGACATTCTGGCGTCTATCGGTGAACCTCCGGTATCAACGCTGGAAGGTGACGCTAACGCAGATGCAGCGAACGCTCGGCGTATTCTCAACAAGATTAACCGACAGAt                                                                                                                                                      <  1:346326/301‑1 (MQ=255)`  
                                                                                                                                                         `ggCCGCACGCACCCGCGCTGCTAACAAAGCCCGAAAGGAAGCTGAGTTGGCTGCTGCCACCGCTGAGCAATAACTAGCATAACCCCTTGGGGCCTCTAAACGGGTCTTGAGGGGTTTTTTGCTGAAAGGAGGAACTATATGCGCTCATGCGATATGAACGTTGAGACTGCCGCTGAGCTATCAGCTGTGAACGACATTCTGGCGTCTATCGGTGAACCTCCGGTATCAACGCTGGAAGGTGACGCTAACGCAGATGCAGCGAACGCTCGGCGTATTCTCAACAAGATTAACCGACAGATTc                                                                                                                                                    <  1:292000/301‑1 (MQ=255)`  
                                                                                                                                                             `gcacgcacCCGCGCTGCTAACAAAGCCCGAAAGGAAGCTGAGTTGGCTGCTGCCACCGCTGAGCAATAACTAGCATAACCCCTTGGGGCCTCTAAACGGGTCTTGAGGGGTTTTTTGCTGAAAGGAGGAACTATATGCGCTCATGCGATATGAACGTTGAGACTGCCGCTGAGGTATCAGCTGTGAACGACATTCTGGCGTCTATCGGTGAACCTCCGGTATCAAAGCTGGAAGGTGACGCTAACGCAGATGCAGCGAACGCTCGGCGTATTCTCAACAAGATTAACCGACAGATTCAAtc                                                                                                                                                >  1:626792/1‑301 (MQ=255)`  
                                                                                                                                                                `cgcacCCGCGCTGCTAACAAAGCCCGAAAGGAAGCTGAGTTGGCTGCTGCCACCGCTGAGCAATAACTAGCATAACCCCTTGGGGCCTCTAAACGGGTCTTGAGGGGTTTTTTGCTGAAAGGAGGAACTATATGCGCCCATGCGATATGAACGTTGAGACTGCCGCTGAGTTATCAGCTGTGAACGACATTCTGGCGTCTATCGGTGAACCTCCGGTATCAACGCTGGAAGGTGACGCTAACGCAGATGCAGCGAACGCTCGGCGTATTCTCAACACGATTAACCGACCGATTCAATCTCg                                                                                                                                             >  1:140348/1‑301 (MQ=255)`  
                                                                                                                                                                  `cacTCGCGCTGCTAACAAAGCCCGAAAGGAAGCTGAGTTGGCTGCTGCCACCGTTGAGCAATAAATAGCATAACCTCTTGGGGCCTCCAAACGGGTCTTGAGGGGTTTTTTGCTGAAAGGAGGAACTATAAGCGCTCACGCGATATGAACGTTGAGCCTGCCGCTGAGTTATCAGCTGTGAACGACATTCTGGCGTCTATCGGTGAACCTCCGGTATCAACGCTGGAAGGTGACGCTAACGCAGATGCAGCGAACGCTCGGCGTATTCTCAACAAGATTAACCGACAGATTCAATCTCGtg                                                                                                                                           >  1:19471/1‑301 (MQ=255)`  
                                                                                                                                                                       `gcgcTGCTAAAAAAGCCCGAAAGGAAGCTGAGTTGGCTGCTGCCACCGCTGAGCAATAACTAGCATAACCCCTTGGGGCCTCTAAACGGGTCTTGAGGGGTTTTTTGCTGAAAGGAGGAACTATATGCGCTCATGCGATATGAACGTTGAGACTGCCGCTGAGTTATCAGCTGTGAACGACATTCTGGCGTCTATCGGTGAACCTCCGGTATCAACGCTGGAAGGTGACGCTAACGCAGATGCAGCGAACGCTCGGCGTATTCTCAACAAGATTAACCGACAGATTCAATCTCGtggatgg                                                                                                                                      <  1:545123/301‑1 (MQ=255)`  
                                                                                                                                                                        `cgcTGCTAACAAAGCCCGAAAGGAAGCTGAGTTGGCTGCTGCCACCGCTGAGCAATTACTAGCATAACCCCTTGGGGCCTCTAAACGGGTCTTGAGGGGTTTTTTGCTGAAAGGAGGAACTATATGCGCTCATGCGATATGAACGTTGAGACTGCCGCTGAGTTATCAGCTGTGAACGACATTCTGGCGTCTATCGGTGAACCTCCGGTATCAACGCTGGAAGGTGACGCTAACGCAGATGCAGCGACCGCTCGGCGTATTCTCAACAAGATTAACCGACAGATTCAATCTCGtggatgga                                                                                                                                     >  1:314918/1‑301 (MQ=255)`  
                                                                                                                                                                          `ctgctAACAAAGCCCGAAAGGAAGCTGAGTTGGCTGCTGCCACCGCTGAGCAATAACTAGCATAACCCCTTGGGGCCTCTAAACGGGTCTTGAGGGGTTTTTTGCTGAAAGGAGGAACTATATGCGCTCATGCGATATGAACGTTGAGACTGCCGCTGAGTTATCAGCTGTGAACGACATTCTGGCGTCTATCGGTGAACCTCCGGTATCAACGCTGGAAGGTGACGCTAACGCAGCTGCAGCGAACGCCCGGCGTATTCTCAACAAGATTAACCGACAGATTCAAtctctgtcacttata                                                                                                                                   >  1:431335/1‑290 (MQ=255)`  
                                                                                                                                                                          `aggctAACAAAGGCCGAAAGGAAGCTGAGTTGGCTGCTGCCACCGCTGAGCAATAACTAGCATAACCCCTTGGGGCCTCTAAACGGGTCTTGAGGGGTTTTTTGCTGAAAGGAGGAACTATATGCGCTCATGCGATATGAACGTTGAGACTGCCGCTGAGTTATCAGCTGTGAACGACATTCTGGCGTCTATCGGTGAACCTCCGGTATCAACGCTGGAAGGTGACGCTAACGCAGATGCAGCGAACGCTCGGCGTATTCTCAACAAGATTAACCGACAGATTCAATCTCGTGGATGGACg                                                                                                                                   <  1:401205/299‑1 (MQ=255)`  
                                                                                                                                                                            `gctAACAAAGCCCGAAAGGAAGCTGAGTTGGCTGCTGCCACCGCTGAGCAATAACTAGCATAACCCCTTGGGGCCTCTAAACGGGTCTTGATGGGTTTTTTGCTGAAAGGAGGAACTATATGCGCTCATGCGATATGCACGTTGAGACTGCCGCTGAGTTATCAGCTGTGAACGACATTCTGGCGTCTATCGGTGAACCTCCGGTATCAACGCTGGAAGGTGACGCTAACGCAGATGCAGCGAACGCTCGGCGTATTCTCAACAAGATTAACCGACAGATTCAATCTAGTGGATGGACGtt                                                                                                                                 >  1:286260/1‑301 (MQ=255)`  
                                                                                                                                                                            `gctAACAAAGCCCGAAAGGAAGCTGAGTTGGCTGCTGCCACCGCTGAGCAATAACTAGCATAACCCCTTGGGGCCTCTAAACGGGTCTTGAGGGGTTTTTTGCTGAAAGGAGGCACTATATGCGCTCATGCGATACGAACGTTGAGACTGCCGCTGAGTTATCAGCTGTGAACGACATTCTGGCGTCTATCGGTGAACCTCCGGTATCAACGCTGGAAGGTGACGCTAACGCAGATGCAGCGAACGCTCGGCGTATTCTCAACAAGATTAACCGACAGATTCAATCTCGTGGATGGACGtt                                                                                                                                 >  1:53366/1‑301 (MQ=255)`  
                                                                                                                                                                            `gctAACAAAGCCCGAAAGGAAGCTGAGTTGGCTGCTGCCACCGCTGAGCAATAACTAGCATAACCCCTTGGGGCCTCTAAACGGGTCTTGAGGGGTTTTTTGCTGAAAGGAGGAACTATATGCGCTCATGCGATATGAACGTTGAGACTGCCGCTGAGTTATCAGCTGTGAACGACATTCTGGCGTCTATCGGTGAACCTCCGGTATCAACGCTGGAAGGTGACGCTAACGCAGATGCAGCGAACGCTCGGCGTATTCTCAACAAGATTAACCGACAGATTCAATCTCGTGGATGGACGtt                                                                                                                                 >  1:272104/1‑301 (MQ=255)`  
                                                                                                                                                                               `aaCAAAGCCCGAAAGGAAGCTGAGTTGGCTGCTGCCACCGCTGAGCAATAACTAGCATAACCCCTTGGGGCCTCTAAACGGGTCTTGAGGTGTTTTTTGCTGAACGGAGGCACTATATGCGCTCATGCGATATGAACGTTGAGACTTCCGCTGAGTTATTAGATGTGAACTACATTCTTGCGTCTATCGGTGAACATCCGGTATCAACGCTGGAAGGTGAGCCTAACGCAGATGCAGCGAACTCTCGGCGTATTCTCAAGAAGATTAACCGACACATTCAATTTCGTTGATGGACGTTCaa                                                                                                                              >  1:424408/1‑301 (MQ=255)`  
                                                                                                                                                                                       `ccGAAAGGAAGCTGAGTTGGCTGCTGCAACCGCTGAGCAATAACTAGAATAAACCCTTGGGGCCTCTAAACGGGGCTTGAGGGGTTTTTTGCGGAAAGGAGGAACTATATGCGCTCATGCGATATGAACGTTGAGACTGCCGCTGAGTTATCAGCTGTGAACGACATTCTGGCGTCTATCGGTGAACCTACGGTATCAACGCTGGAAGGTGACGCTAACGCAGATGCAGCAAACGCTCGGCGTATTCTCAACAAGATTAACCGACAGATTCAATCTAGTGGATGGACGTTCAACATTGAgg                                                                                                                      <  1:73902/301‑1 (MQ=255)`  
                                                                                                                                                                                       `ccGAAAGGAAGCTGAGTTGGATGGTGCCACCGCTGAGCAATAACTAGCATAACCACGTGGGGCCTCTAAACGGGTCGTGAGGGGTTTTTTGCTGAAAGGAGGAACTATATGCGATCATGCGATATGAACGTTGAGACTGCCGCTGAGTTATCAGCTGTGAACGACATTCAGGCGTCTATCGGTGAACCTCCGGTATCAACGCTGGAAGGTGACGCTAACGCAGATGCAGCGAACGCTCGGCGTATTCTCAACAAGATTAACCGACAGATTCAATCTCGGGGATGGACGTTCAACATTGAgg                                                                                                                      <  1:32660/301‑1 (MQ=255)`  
                                                                                                                                                                                                    `gAGTTGGCTGCTGCCACCGCTGAGCAATAACTAGCATAACCCCTTGGGGCCTCTAAACGGGTCTTGAGGGGTTTTTTGCTGAAAGGAGGAACTATATGCGCTCATGCGATATGAACGTTGAGACTGCCGCTGAGTTATCAGCTGTGAACTACATTCTGGCGTCTATCGGTGAACATCCGGTATCAACGCTGGAAGGTTACGCTAATGCAGATGCAGCGAACGCTCGTCGTCTTCTCAACAAGATTAACCGCCAGATTCACTCTCGTGGATGGACGTTCAACATTGCGGAAGGCATAACGct                                                                                                         >  1:619631/1‑301 (MQ=255)`  
                                                                                                                                                                                                       `ttGGCTGCTGCCACCTCTGAGCAATAACTAGCATAACCCCTTGGGGCCTCTAAACGGGTCTTGAGGGGTTTTTTGCTGAAAGGAGGAACTATATGCGCTCATGCGATATGAACGTTGAGACTGCAGCTGAGTTATCAGCTGTGAACGACATTCTGGCGTCTATCGGTGAACCTCCGGTATCAACGCTGGAAGGTGACGCTAACGCAGATGCAGCGAACGCTCGGCGTATTCTCAACAAGATTAACCGACAGATTCAATCTCGTGGATGGACGTTCAACATTGAGGAAGGCATAACGctact                                                                                                      <  1:14717/301‑1 (MQ=255)`  
                                                                                                                                                                                                          `gctgcgGCAACCGCTGGGGAATAACTAGAATAAGCACTGGGGGCATATAAACGGTTCTTGAAGGGTTTTTTGCTGAAAAGAGGAACGATATGCGGTCATGCGATATGAACGTTGAGACTGCCGCTGAGTTATCAGCTGTGAACGACATTCTGGAGTCTATCGGTGAACCTCCGGTATCAACGCTGGAAGGTGACGCTAACGCAGATGCAGAGAACGCTCGGCGTATTCTCAACAAGATTAACCGACAGATTCAATCTCGTGGATGGACGTTCAACATTGAGGAAGGCATAACGCTACTAcc                                                                                                   <  1:406902/301‑1 (MQ=255)`  
                                                                                                                                                                                                           `acagtgcCACCGCTGAGCAATAACTAGCATGACCCCTTGGGGCCTCTAAACGGGTCTTGAGGGGTTTTTTGCTGAAAGGAGGAACTATATGCGCTCATGCGATATGAACGTTGAGACTGCCGCTGAGTTATCAGCTGTGAACGACATTCTGGCGTCTATCGGTGAACCTCCGGTATCAACGCTGGAAGGTGACGCTAACGCAGATGCAGCGAACGCTCGGCGTATTCTCAACAAGATTAACCGACAGATTCAATCTCGTGGATGGACGTTCAACATTGAGGAAGGCATAACGCTACTACCt                                                                                                  <  1:570386/297‑1 (MQ=255)`  
                                                                                                                                                                                                               `tgcCACCGCTGAGCAATAACTAGCATAACCCCTTGGGGCCTCTAAACGGGTCTTGAGGGGTTTTTTGCTGAAAGGAGGAACTATATGCGCTCATGCGATATGAACGTTGAGACTGCCGCTGAGTTATCAGCTGTGAACGACATTCTGGCGTCTATCGGTGAACCTCCGGTATCAACGCTGGAAGGTGACGGTAACGCAGATGCAGCGAACGCTCGGCGTATTCTCAACAAGATTAACCGACAGATTCAATCTCGTGGATGGACGTTCAACATTGAGGAAGGCATAACGCTACTACCTGATg                                                                                              <  1:27960/301‑1 (MQ=255)`  
                                                                                                                                                                                                                    `ccGCTGAGCAATAACTAGCATAACCCCTTGGGGCCTCTAAACGGGTCTTGAGGGGTTTTTTGCTGAAAGGAGGAACTATATGCGCTCATGCGATATGAACGTTGAGACTGCCGCTGAGTTATCAGCTGTGAACGACATTTTGGCGTCTATCGGTGAATCTCCGGTATCAACGCTGGAAGGTGACGCTAACGCAGATGCAGCGAACGATCGGCGTATTCTCAACAAGATTAACCGACAGATTCAATTTCGTGGATGGACGTTCAACATTGAGGAAGGCATAACGCTACTACCTGAAGTTTat                                                                                         >  1:507931/1‑300 (MQ=255)`  
                                                                                                                                                                                                                    `ccGCTGAGAAATAACTAGCATAAACCCTTGGGGCCTCTAAAAGGGGCTTGAGGGGGTTGTTGCTGAAAGGAGGAACTATATGAGCTAATGCGATATGAACGGTGAGACTGCCGCTGAGTTATCAGCTGTGAACGACATTCTGGAGTCTATCGGTGAACAGCCGGTATCAACGCTGGAAGGTGACGCTAACGCAGATGCAGCGAACGCTCGGCGTATTCTCAACAAGATTAACCGACAGATTCAATCTCGTGGATGGACGTTCAACATTGAGGAAGGCATAACGCTACTACCTGATGTTTAc                                                                                         <  1:302440/301‑1 (MQ=255)`  
                                                                                                                                                                                                                         `gAGCAATAACTAGCATAACCCCTTGGGGCCTCT‑AACGGGTCTTGAGGGGTTTTTTGCTGAAAGGAGGAACTATATGCGCTCATGCGATATGAACGTTGAGACTGCCGCTGAGTTATCAGCTGTGAACGACATTCTGGCGTCTATCGGTGAACCTCCGGTATCAACGCTGGAAGGTGACGCTAACGCAGATGCAGCGAACGCTCGGCGTATTCTCAACAAGATTAACCGACAGATTCAATCTCGTGGATGGACGTTCAACATTGAGGAAGGCATAACGCTACTACCTGATGTTTACTCCAAc                                                                                   >  1:93503/1‑301 (MQ=255)`  
                                                                                                                                                                                                                              `aTAACTAGCATAACCCCTTGGGGCCTCTAAACGGGTCTTGAGGGGTTTTTTGCTGAAAGGAGGAACTATATGCGCTCATGCGATATGAACGTTGAGACTGCCGCTGAGTTATCAGCTGTGAACGACATTCTGGCGTCTATCGGTGAACCTCCGGTATCAACGCTGGAAGGTGACGCTAACGCAGATGCAGCGAACGCTCGGCGTATTCTCAACAAGATTAACCGACAGATTCAATCTCGTGGATGGACGTTCAACATTGAGGACGGCATAACGCTACTACCTGATGtctttctcttataca                                                                               >  1:417864/1‑287 (MQ=255)`  
                                                                                                                                                                                                                               `aaaCTAGCATAACCCCGTGGGGCCTCGAAACGGGTCTTGAGGGGTTTTTTGCTGAAAGGAGGAACTATATGCGCTCATGCGATATGAACGTTGAGACTGCCGCTGAGTTATCAGCTGTGAACGACATTCTGGCGTCTATCGGTGAACCTCCGGTATCAACGCTGGAAGGTGACGCTAACGCAGATGCAGCGAACGCTCGGCGTATTCTCAACAAGATTAACCGACAGATTCAATCTCGTGGATGGACGTTCAACATTGAGGAAGGCATAACGCTACTACCTGATGTTTACTCCAACCTGAt                                                                              <  1:412543/300‑1 (MQ=255)`  
                                                                                                                                                                                                                                    `cgtgggctcggagaagtgtataagagacagcTTGAGGGGTTTTTTGCTGAAAGGAGGAACGATATGCGCTCATGCGATAGTAACGTTGAGACTGCCGCTGAGTTATCAGCTGTGAACGACATTCTGGCGTCTATCGGTGAACCTCCGGTATCAACGCTGGAAGGTGACGCTAACGCAGAAGCAGCGAACGCTCGGCGTATTCTCAACAAGATTAACCGACAGATTCAATCTCGTGGATGGACGTTCAACATTGAGGAAGGCATAACGCTACTACCTGATGTTTACTCCAACCTGATTGtat                                                                         <  1:376761/271‑1 (MQ=255)`  
                                                                                                                                                                                                                                    `aGCATAACCCCTTGGGGCCTCTAAACGGGTCTTGAGGGGTTTTTTGCTGAAAGGAGGAACTATATGCGCTCATGCGATATGAACGTTGAGACTGCCGCTGAGTTATCAGCTGTGAACGACATTCTGGCGTCTATCGGTGAACCTCCGGTATCAACGCTGGAAGGTGACGCTAACGAAGCTGCAGCGAACGCTCGGCGTATTCTCAACAAGATTAACCGACAGATTCAATCTCGTGGATGGACGTTCAACATTGAGGAAGGCATAACGCTACTACCTGATGTATAATccctgtctcttataa                                                                         >  1:649333/1‑288 (MQ=255)`  
                                                                                                                                                                                                                                     `gCATAACCCCTTGGGGCCTCTAAACGGGTCTTGAGGGGTTTTTTGCTGAAAGGAGGAACTATATGCGCTCATGCGATATGAACGTTGAGACTGCCGCTGAGTTATCAGCTGTGAACGACATTCTGGCGTCTATCGGTGAACCTCCGGTATCAACGCTGGAAGGTGACGCTAACGCAGATGCAGCGAACGCTCGGCGTATTCTCAACAAGATTAACCGACAGATTCAATCTCGTGGATGGACGTTCAACATTGAGGAAGGCATAACGCTACTACCTGATGTTTACTCCAACCTGATTGtata                                                                        >  1:249241/1‑301 (MQ=255)`  
                                                                                                                                                                                                                                     `gCATAACCCCTTGGGGCCTCTAAACGGGTCTTGAGGGGTTTTTTGCTGAAAGGAGGAACTATATGCGCTCATGCGATATGAACGTTGAGACTGCCGCTGAGTTATCAGCTGTGAACGACATTCTGGCATCTATCGGTGAACCTCCGGTATCAACGCTGGAAGGTGACGCTAACGCAGATGCAGCGAACGCCCGGCGTATTCTCAACAAGATTAACCGACAGATTCAATCTCGTGGATGGACGTTCAACATTGAGGAAGGCATAACGCTACTACCTGATGTTTACTCCAACCTGATTGtata                                                                        >  1:134180/1‑301 (MQ=255)`  
                                                                                                                                                                                                                                      `gatgtgtataagagacggctAAACGGGGTTTGAGGGGTTTTTTGCTGAAAGGAGGAACTATATGCGCTCATGCGATATGAACGTTGAGACTGCCGCTGAGTTATCAGCTGTGAACGACATTCTGGCGTCTATCGGTGAACCTCCGGTATCAACGCTGGAAGGTGACGCTAACGCAGATGCAGCGAACGCTCGGCGTATTCTCAACAAGATTAACCGACAGATTCAATCTCGTGGATGGACGTTCAACATTGAGGAAGGCATAACACTACTACCTGATGTTTACTCCAACCTGATTGTATAc                                                                       <  1:112680/283‑1 (MQ=255)`  
                                                                                                                                                                                                                                      `gacagccccTTGGGGCCTCTAAACGGGTCTTGAGGTGTTTTGTGCTGAAAGGAGGAACTATATGCGCTCATGCGATATGAACGTTGAGACTGCCGCTGAGTTATCAGCTGTGAACGACAGTCTGGCGTCTATCGGTGAACCTCCGGTATCAACGCTGGAAGGTGACGCTAACGCAGATGCAGCGAACGCTCGGCGTATTCTCAACAAGATTAACCGACAGATTCAATCTCGTGGATGGACGTTCAACATTGAGGAAGGCATAACGCTACTACCTGATGTTTACTCCAACCTGATTGTATAc                                                                       <  1:539539/296‑1 (MQ=255)`  
                                                                                                                                                                                                                                      `gacagccccTTGGGGCCTCTAAACGGGTCTTGAGGGGTTTTTTGCTGAAAGGAGGAACTATATGCGCTCATGCGATATGAACGTTGAGACTGCCGCTGAGTTATCAGCTGTGAACGACATTCTGGCGTCTATCGGTGAACCTCCGGTATCAACGCTGGAAGGTGACGCTAACGCAGATGCAGCGAACGCTCGGCGTATTCTCAACAAGATTAACCGACAGATTCAATCTCGTGGATGGACGTTCAACATTGAGGAAGGCATAACGCTACTACCTGATGTTTACTCCAACCTGATTGTATAc                                                                       <  1:521380/296‑1 (MQ=255)`  
                                                                                                                                                                                                                                      `cATGACCCCTTGGGGCCTCTAAACGGGTCTTGAGGGGTTTTTTGCTGAAAGGAGGAACTATATGCGCTCAGGCGATATGAACGTGGAGACTGCCGCTGAGTTATCAGCTGTGAACGACATTCTGGCGTCTATCGGTGAACCTCCGGTATCAACGCTGGAAGGTGACGCTAACGCAGATGCAGCGAACGCTCGGAGTATTCTCAACAAGATTAACCGACAGATTCAATCTCGTGGATGGACGTTCAACATTGAGGAAGGCATAACGCTACTACCTGATGTTTACTCCAACCTGATTGTATAc                                                                       <  1:242731/301‑1 (MQ=255)`  
                                                                                                                                                                                                                                      `cATAACCCCTTGGGGCCTCTAAACGGGTCTTGAGGGGTTTTTTGCTGAAAGGAGGAACTATATGCGCTCATGCGATATGAACGTTGAGACTGCCGCTGAGTTATCAGCTGTGAACGACATTCTGGCGTCTATCGGTGAACCTCCGGTATCAACGCTGGAAGGTGACGCTAACGCAGATGCAGCGAACGCTCGGCGTATTCTCAACAAGATTAACCGACAGATTCAATCTCGTGGATGGACGTTCAACATTGAGGAAGGCATAACGCTACTACCTGATGTTTACTCCAACCTGATTGTATAc                                                                       <  1:591207/301‑1 (MQ=255)`  
                                                                                                                                                                                                                                      `cATAACCCCTTGGGGCCTCTAAACGGGTCTTGAGGGGTTTTTTGCTGAAAGGAGGAACTATATGCGCTCATGCGATATGAACGTTGAGACTGCCGCTGAGTTAGCAGCTGTGAACGACATTCTGGCGTCTATCGGTGAACCTCCGGTATCAACGCTGGAAGGTGACGCTAACGCAGATGCAGCGAACGCTCGGCGTATTCTCAACAAGATTAACCGACAGATTCAATCTCGTGGATGGACGTTCAACATTGAGGAAGGCATAACGCTACTACCTGATGTTTACTCCAACCTGATTGTATAc                                                                       <  1:447439/301‑1 (MQ=255)`  
                                                                                                                                                                                                                                      `aaTAACCCCTTGGGGCCTCTAAACGGGTCTTGAGGGGTTTTTTGCTGAAAGGAGGAACTATATGCGCTAATGCGATATGAACGGTGAGACTGCCGCTGAGTTAGCAGCTGTGAACGACATTCTGGCGTCTATCGGTGAACCTCCGGTATCAACACTGGAAGGTGACGCTAACGCAGATGCAGCGAACGCTCGGCGTATTCTCAACAAGATTAACCGACAGATTCAATCTCGTGGATGGACGTTCAACATTGAGGAAGGCATAACGCTACTACCTGATGTTTACTCCAACCTGATTGTATAc                                                                       <  1:575763/300‑1 (MQ=255)`  
                                                                                                                                                                                                                                        `aaaCCCCTTGGGGCCTCTAAACGGGTCTTGAGGGGTTTTTTTCTGAAAGGAGGAACTATATGCGCTCATGCGATATTAACGTTGAGACTGCCGCTGAGTTATCAGCTGTGAACGACATTCTGGCGTCTATCGGTGAACCTCCGGTATCAACGCTGGAAGGTGACGCTAACGCAGATGCAGCGAACGCTCGGCGTATTCTCAACAAGATTAACCGACAGATTCAATCTCGTGGATGGACGTTCAACATTGAGGAAGGCATAACGCTACTACCTGATGTTTACTCCAACCTGATTGTATACAg                                                                     <  1:370346/300‑1 (MQ=255)`  
                                                                                                                                                                                                                                            `cccTTGGGGCCTCTAAACGGGTCTTGGGGGGTTTTTTGCTGAAAGGAGGAACTATATGCGCTCATGCGATATGAACGTTGAGACTGCCGCTGAGTTATCAGCTGTGAACGACATTCTGGCGTCTATCGGTGAACCTCCGGTATCAACGCTGGAAGGTGACGCTAACGCAGATGCAGCGAACGCTCGGCGTATTCTCAACAAGATTAACCGACAGATTAAATCCCGTGGATGGACGTTCAACATTGAGGAAGGCATAACGCTACTACCTGATGTTTACTCCAACCTGATTGTATAcctgtct                                                                 >  1:466914/1‑295 (MQ=255)`  
                                                                                                                                                                                                                                            `cccTTGGGGCCTCTAAACGGGTCTTGAGGGGTTTTTTGCTGAAAGGAGGAACTATATGCGCTCATGCGATATGAACGTTGAGACTGCCGCTGAGTTATCATCTGTGAACGACATTCTGGCGTCTATCGGTGAACCTCCGGTATCAACGCTGGAAGGTGACGCTAACGCAGATGCAGCGAACGCCCGGCGTATTCTCAACAAGATTAACCGACAGATTCAATCTCGTGGATGGACGTTCAACATTGAGGCAGGCATAACGCTACTACCTGATGTTTACTCCAACCTGTTTgctgtcttttat                                                                 >  1:557231/1‑290 (MQ=255)`  
                                                                                                                                                                                                                                                    `gCCTCTAAACGGGTCTTGAGGGGTTTTTTGCTGAAAGGAGGAACTATATGCGCTCATGCGATATGAACGTTGAGACTGCCGCTGAGTTATCAGCTGTTAACGACATTCTGGCGTCTATCGGTGAACCTCCGGTATCAACGCTGGAAGGTGACGCTAACGCAGATGCAGCGAACGCTCGGCGTATTCTCAACAAGATTAACCGACAGATTCAATCTCGTGGATGGACGTTCAACATTGAGGAAGGCATAACGCTACTACCTGATGTTTACTCCAACCTGATTGTATACAGTGACGACTAttt                                                         <  1:182754/301‑1 (MQ=255)`  
                                                                                                                                                                                                                                                        `ctAAACGGGTCTGGAGGGGTTTTTTGCTGAAAGGAGGAACTATATGCGCTCATGCGATATGAACGTTGAGACTGCCGCTGAGTTATCAGCTGTGAACGACATTCTGGCGTCTATCGGTGAACCTCCGGTATCAACGCTGGAAGGTGACGCTAACGCAGATGCCGCGAACGCTCGGCGTATTCTCAACAAGATTAACCGACAGATTCAATCTCGTGGATGGACGTTCAACATTGAGGAAGGCATAACGCTACTACCTGATGTTTACTCCAACCTGATTGTATACAGTGACGACTATTTATcc                                                     <  1:666434/301‑1 (MQ=255)`  
                                                                                                                                                                                                                                                               `ggTCTTGAGGGGTTTTTTGCTGAAAGGAGGAACTATATGCGCTCATGCGATATGAACGTTGAGACTGCCGCTGAGTTATCAGCTGTGAACGACATTCTGGCGTCTATCGGTGAACCTCCGGTATCAACGCTGGAAGGTGACGCTAACGCAGATGCAGCGAACTCTCGGCGTATTCTCAACAAGATTAACCGACAGATTCAATCTCGTGGATGGACGTTCAACATTGAGGAAGGCATAACGCTACTACCTGATGTTTACTCCAACCTGATTGTATACAGTGACGACTATTTATCCCTAATGt                                              >  1:222077/1‑301 (MQ=255)`  
                                                                                                                                                                                                                                                                      `gcggagatgtgtagaagagacagaACGATATGCGCTCATGCGATATGAACGTGGAGACTGCCGCTGAGTTATCAGCTGGGAACGACATTCTGGCGTCTATCGGTGAACCTCCGGTATCAACGCTGGAAGGGGACGCTAACGCAGATGCAGCGAACGCTCGGCGTATTCTCAACAAGATGAACCGACAGATTCAATCTCGTGGATGGACGTTCAACATTGAGGAAGGCATAACGCTACTACCTGATGTTTACTCCAACCTGATTGTATACAGTGACGACTATTTATCCCTAATGTCTACTTc                                       <  1:333626/279‑1 (MQ=255)`  
                                                                                                                                                                                                                                                                      `aGGGGTTTTTTGCTGAAAGGAGGCACTATATGCGCTCATGCGATATGAACGTTGAGACTGCCGCTGAGTTATCAGCTGTGAACGACATTCTGGCGTCTATCGGTGAACCTCCGGTATCAACGCTGGAGGGTGACGCTAACGCAGATGCAGCGAACGCTCGGCGTATTCTCAACAAGATTAACCTACAGATTCAATCTCGTGGATTGACGTTCAACATTGAGGAAGGCATAACGCTACTACCTGATGTTTACTCCAACCTGATTGTATACAGTGACGACTATTTATCCCTAATGTCTACTTc                                       <  1:392999/301‑1 (MQ=255)`  
                                                                                                                                                                                                                                                                          `gTTTTTTGCTGAAAGGAGGAACTATATGCGCTCATGCGATATGAACGTTGAGACTGCCGCTGAGTTATCAGCTGTGAACGACATTCTGGCGTCTATCGGTGAACCTCCGGTATCAACGCTGGAAGGTGACGCTAACGCAGATGCAGCGAACGCTCGGCGTATTCTCAACAAGATTAACCGACAGATTCAATCTCGTGGATGGACGTTCAACATTGAGGAAGGCATAACGCTACTACCTGATGTTTACTCCAACCTGATTGTATACAGTGACGACTATTTATCCCTAATGTCTACTTCCGGt                                   <  1:255491/301‑1 (MQ=255)`  
                                                                                                                                                                                                                                                                          `gTTTTTTGCTGAAAGGAGGAACTATATGCGCTCATGCGATATGAACGTTGAGACTGCCGCTGAGTTATCAGCTGTGAACGACATTCTGGCGTCTATCGGTGAACCTCCGGTATCAACGCTGGAAGGTGACGCTAACGCAGATAAAGCGAACGCTCGGCGTATTCTCAACAAGATTAACCGACAGATTCAATCTCGTGGATGGACGTTCAACATTGAGGAAGGCATAACGCTACTACCTGATGTTTACTCTAACCTGATTGTATACAGAGACGACTATTTATCCCTAATgctgtctcttatt                                   >  1:661468/1‑289 (MQ=255)`  
                                                                                                                                                                                                                                                                           `ttttttGCTGAAAGGAGGAACTATATGCGCTCATGCGATATGAACGTTGAGACTGCCGCTGAGTTATCAGCTGTGAACGACATTCTGGCGTCTATCGGTGAACCTCCGGTATCAACGCTGGAAGGTGACGCTAACGCAGATGCAGCGAACGCTCGGCGTATTCTCAACAAGATTAACCGACAGATTCAATCTCGTGGATGGACGTTCAACATTGAGGAAGGCATAACGCTACTACCTGATGTTTACTCCAACCTGATTGTATACAGTGACGACTACTTATCCCTAATGTCTCCTTcctttc                                  >  1:122338/1‑297 (MQ=255)`  
                                                                                                                                                                                                                                                                           `ttgtttGCTGAAAGGAGGAACTAGATGCGCTCATGCGAGATGAACGTTGAGACCGCCGCTGAGTTATCAGCTGTGAACGACATTCGGGCGTCTATCGGTGAACCTCCGGTATCAACGCTGGAAGGTGACGCCAACGCAGATGCAGCGAACGCTCGGCGTATTCTCAACAAGATTAACCGACAGATTCAATCTCGTGGATGGACGTTCAACATTGAGGAAGGCATAACGCTACTACTTGATGTTTACTCCAACCTGATTGTATACAGTGACGACTATTTATCCCTAATGTCTACTTCCGGTc                                  <  1:359235/298‑1 (MQ=255)`  
                                                                                                                                                                                                                                                                            `tttttGCTGAAAGGAGGAACTATATGCGCTCATGCGATATGAACGTTGAGACTGCCGCTGAGTTATCAGCTGTGAACGACATTCTGGCGTCTATCGGTGAACCTCCGGTATCAACGCTGGAAGGTGACGCTAACGCAGATGCAGCGAACGCTCGGCGTATTCTCAACAAGATTAACCGACAGATTCAATCTCGTGGATGGACGTTCAACATTGAGGAAGGCATAACGCTACTACCTGATGTTTACTCCAACCTGATTGTATACAGTCACGACTATTTATCCCTAATGTCTACTTCCGGTCa                                 >  1:460565/1‑301 (MQ=255)`  
                                                                                                                                                                                                                                                                                `aagtgccatgaggaACATTAGGCGCCCATTCGAAATTAGCGTTGGTACCGCCTCCTAGTTAACAGCTGTGAACGACATTCTGGCGTCTATCGTTTAACCTCCTGTATCAACGCTTGAAGGTGACGCTAACGCAGATGCAGCGAACGCTCGGCAGATTCTCAACAAGATTAACCGACAGATTCAATCTTGTGGATGGACGTTCAACATTTAGGAAGGCATAACGCTACTACCTGATGTTTACTCCAACCTGATTGGATACAGTGACGACTATTTATCCCTAATGTCTACTTCCGGTCAATcc                             <  1:106027/292‑1 (MQ=255)`  
                                                                                                                                                                                                                                                                                   `agacaggaggAACTATATGCGCTCATGCGATATGAACGTTGAGACTGCCGCTGAGTTATCAGCTGTGAACGACATTCTGGCGTCTATCGGTGAACCTCCGGTATCAACGCTGGAAGGTGACGCTAACGCAGATGCAGCGAACGCTCGGCGTATTCTCAACAAGATTAACCGACAGATTCAATCTCGTGGATGGACGTTCAACATTGAGGAAGGCATAACGCTACTACCTGATGTTTACTCCAACCTGATTGTATACAGTGACGACTATTTATCCCTAATGTCTACTTCCGGTCAATCCATc                          <  1:454540/297‑1 (MQ=255)`  
                                                                                                                                                                                                                                                                                     `aaaGGAGGAACTATATGCGCTCAGGGGATATGAACGTTGAGACTGCCGCTGAGTTATCAGCTGTGAACGACATTCTGGCGTCTATCGGTGAACCTCCGGTATCAACGCTGGAAGGTGACGCTAACGCAGATGCAGCGAACGCTCGGCGTATTCTCAACAAGATTAACCGACAGATTCAATCTCGTGGATGGACGTTCAACATTGAGGAAGGCATAACGCTACTACCTGATGTTTGCTCCAACCTGATTGTATACAGTGACGACTATTTATCCCTAATGTCTACTTCCGGTCAATCCATCTa                        <  1:8706/301‑1 (MQ=255)`  
                                                                                                                                                                                                                                                                                        `ggaggacagatattaggtCATGCGCTATGAACGTTGTGACTGCCGCTTGATTATCAGCTGTGAACGTCATTCTGGCGTCTATCGGTGAACCTCCGGTAACAACGCTGGAAGGTGACGCTAACGCAGATGCTGCGAGCGCTCGGCGTATTCTCAACAAGATTAACCGACAGATTCAATCTCGTAGATGGACGTCCAACATTGAGGAAGGCAGAACGCTACTACCTGATGTTTACTCCAACCTGATTGTATACACTGACGACTATTTATCCCTAATGTCTACTTCCGGTCAATCCATCTACGt                     <  1:437473/284‑1 (MQ=255)`  
                                                                                                                                                                                                                                                                                        `aagagacagttatGCGCTCATGCGATATGAACGTTGAGACTGCCGCTGAGTTATCAGCTGTGAACGACATTCTGGCGTCTATCGGTGAACCTCCGGTATCAACGCTGGAAGGTGACGCTAACGCAGATGCAGCGAACGCTCGGCGTATTCTCAACAAGATTAACCGACAGATTCAATCTCGTGGATGGACGTTCAACATTGAGGAAGGCATAACGCTACTACCTGATGTTTACTCCAACCTGATTGTATACAGTGACGACTATTTATCCCTAATGTCTACTTCCGGTCAATCCATCTACGt                     <  1:261456/291‑1 (MQ=255)`  
                                                                                                                                                                                                                                                                                         `gaggaACTATATGCGCTCATGCGATATGAACGTTGAGACTGCCGCTGAGTTATCAGCTGTGAACGACATTCTGGCGTCTATCGGTGAACCTCCGGTATCAACGCTGGACGGTGACGCTAACGCAGATGCAGCGAACGCTCGGCGTCTTCTCAACAAGATTAACCGACAGATTCAATCTCGTGGATGGACGTTCAACATTGAGGAAGGCATAACGCTACTACCTGATGTTTACTCCAACCTGATTGTATACAGTGACGACTATTTATCCCTAATGTCTACTTCCGGTTAATCCATCTACGtt                    >  1:1895/1‑301 (MQ=255)`  
                                                                                                                                                                                                                                                                                             `aaCTATATGCGCTCATGCGATATGAACGTTGAGACTGCCGCTGAGTTATCAGCTGTGAACGACATTCTGGCGTCTATCGGTGAACCTCCGGTATCAACGCTGGAAGGTGACGCTAACGCAGATGCAGCGAACGCTCGGCGTATTCTCAACAAGATTAACCGACAGATTCAATCTCGTGGATGGACGTTCAACATTGAGGAAGGCATAACGCTACTACCTGATGTTTACTCCAACCTGATTGTATACAGTGACGACTATTTATCCCTAATGTCTACTTCCGGTCAATCCATCTACGTTAAcc                >  1:158956/1‑301 (MQ=255)`  
                                                                                                                                                                                                                                                                                              `aCTATATGCGCTCATGCGATATGAACGTTGAGACTGCCGCTGAGTTATCAGCTGTGAACGACATTCTGGCGTCTATCGGTGAACCTCCGGTATCAACGCTGGAAGGTGACGCTAACGCAGATGCAGCGAACGCTCGGCGTATTCTCAACAAGATTAACCGACAGATTCAATCTCGTGGATGGACGTTCAACATTGAGGAAGGCATAACGCTACTACCTGATGTTTACTCCAACCTGATTGTATACAGTGACGACTATTTATCCCTAATGTCTACTTCCGGTCAATCCATCTACGTTAACCg               <  1:607946/301‑1 (MQ=255)`  
                                                                                                                                                                                                                                                                                               `cTATATGCGCTCATGCGATATGAACGTTGAGACTGCCGCTGAGTTATCAGCTGTGAACGACATTCTGGCGTCTATCGGTGAACCTCCGGTATCAACGCTGGAAGGTGACGCTAACGCAGATGCAGCGAACGCTCGGCGTATTCTCAACAAGATTAACCGACAGATTCAATCTCGTGGATGGACGTTCAACATTGAGGAAGGCATAACGCTACTACCTGATGTTTACTCCAACCTGATTGTATACAGTGACGACTATTTATCCCTAATGTCTACTTCCTGTCACTCCATCTACGTTAACCGa              >  1:164744/1‑301 (MQ=255)`  
                                                                                                                                                                                                                                                                                               `cTATATGCGCTCATGCGATATGAACGTTGAGACTGCCGCTGAGTTATCAGCTGTGAACGACATTCTGGCGTCTATCGGTGAACCTCCGGTATCAACGCTGGAAGGTGACGCTAACGCAGATGCAGCGAACGCTCGGCGTATTCTCAACAAGATTAACCGACAGATTCAATCTCGTGGATGGACGTTCAACATTGAGGAAGGCATAACGCTACTACCTGATGTTTACTCCAACCTGATTGTATACAGTGACGACTATTTATCCCTAATGTCTACTTCCGGTCAATCCATCTACGTTAACCGa              >  1:528820/1‑301 (MQ=255)`  
                                                                                                                                                                                                                                                                                                 `atatGCGCTCATGCGATATGAATGTTGAGACTGCCGCTGAGTTATCAGCTGTGAACGACATTCTGGCGTCTATCGGTGAACCTCCGGTATCAACGCTGGAAGGTGACGCTAACGCAGATGCAGCGAACGCTCGGCGTATTCTCAACAAGATTAACCGACAGATTCAATCTCGTGGATGGACGTTCAACATTGAGGAAGGCATAACGCTACTACCTGATGTTTACTCCAACCTGATTGTATACAGTGACGACTATTTATCCCTAATGTCTACTTCCGGTCAATCCATCTACGTTAACCGAgg            >  1:601658/1‑301 (MQ=255)`  
                                                                                                                                                                                                                                                                                                 `atatGCGCTCATGCGATATGAACGTTGAGACTGCCGCTGAGTTATCAGCTGTGAACGACATTCTGGCGTCTATCGGTGAACCTCCGGTATCAACGCTGGAAGGTGACGCTAACGCAGATGCAGCGAACGCTCGGCGTATTCTCAACAAGATTAACCGACAGATTCAATCTCGTGGATGGACGTTCAACATTGAGGAAGGCATAACGCTACTACCTGATGTTTACTCCAACCTGATTGTATACAGTGACGACTATTTATCCCTAATGTCTACTTCCGGTCAATCCATCTACGTTAACCGAgg            >  1:636867/1‑301 (MQ=255)`  
                                                                                                                                                                                                                                                                                                 `atatGCGCTCATGCGATATGAACGTTGAGACTGCCGCTGAGTTATCAGCTGTGAACGACATTCTGGCGTCTATCGGTGAACCTCCGGTATCAACGCTGGAAGGTGACGCTAACGCAGATGCAGCGAACGCTCGGCGTATTCTCAACAAGATTAACCGACAGATTCAATCTCGTGGATGGACGTTCAACATTGAGGAAGGCATAACGCTACTACCTGATGTTTACTCCAACCTGATTGTATACAGTGACGACTATTTATCCCTAATGTCTACTTCCGGTCAATCCATCTACGTTAACCGAgg            >  1:642936/1‑301 (MQ=255)`  
                                                                                                                                                                                                                                                                                                   `atGCGATCATGCGATATGAACGTTGAGACTGCCGCTGAGTTATCAGCTGTGAACGACATTCTGGCGTCTATCGGTGAACCTCCGGTATCAACGCTGGAAGGTGACGCTAACGCAGATGCAGCGAACGCTCGGCGTATTCTCAACAAGATTAACAGACAGATTCAATCTCGTGGATGGACGTTCAACATTGAGGAAGGCATAACGCTACTACCTGATGTTTACTCCAACCTGATTGTATACAGTGACGACTATTTATCCCTAATGTCTACTTCCGGTCAATCCATCTACGTTAACCGAGGTg          <  1:486245/301‑1 (MQ=255)`  
                                                                                                                                                                                                                                                                                                      `cgcTCATGCGATATGAACGTTGAGACTGCCGCTGAGTTATCAGCTGTGAACGACATTCTGGCGTCTATCGGTGAACCTCCGGTATCAACGCTGGAAGGTGACGCTAACGCAGATGCAGCGAACGCTCGGCGTATTCTCAACAAGATTAACCGACAGATTCAATCTCGTGGATGGACGTTCAACATTGAGGAAGGCATAACGCTACTACCTGATGTTTACTCCAACCTGATTGTATACAGTGACGACTATTTATCCCTAATGTCTACTTCCGGTCAATCCATCTACGTTAAccctgtctctt       >  1:152744/1‑292 (MQ=255)`  
                                                                                                                                                                                                                                                                                                       `cagcATGCGATATGAACGTTGAGACTGCCGCTGAGTTATCAGCTGTGAACGACATTCTGGCGTCTATCGGTGAACCTCCGGTATCAACGCTGGAAGGTGACGCTAACGCAGATGCAGCGAACGCTCGGCGTATTCTCAACAAGATTAACCGACAGATTCAATCTCGTGGATGGACGTTCAACATTGAGGAAGGCATAACGCTACTACCTGATGTTTACTCCAACCTGATTGTATACAGTGACGACTATTTATCCCTAATGTCTACTTCCGGTCAATCCATCTACGTTAACCGAGGTGGCTa      <  1:67965/298‑1 (MQ=255)`  

                                                                                                                                                                                                                                                                                                             `|`                                                                                                                                                                                                                                                                                                            
`GTACGCAATGGGCCACGGTGGTCTTCGCCCAGAAGCTGCTGGTGCAGTGGTTTTCAAAGTGGAGTAATGC‑TGGGGGTGGCCTCAACGGTCGCTGCTAGTCCCGAAGAGGCGAGTGTTACTTCAACAGAAGAAACCTTAACGCCAGCACAGGAGGCCGCACGCACCCGCGCTGCTAACAAAGCCCGAAAGGAAGCTGAGTTGGCTGCTGCCACCGCTGAGCAATAACTAGCATAACCCCTTGGGGCCTCTAAACGGGTCTTGAGGGGTTTTTTGCTGAAAGGAGGAACTATATGCGCTCATACGATATGAACGTTGAGACTGCCGCTGAGTTATCAGCTGTGAACGACATTCTGGCGTCTATCGGTGAACCTCCGGTATCAACGCTGGAAGGTGACGCTAACGCAGATGCAGCGAACGCTCGGCGTATTCTCAACAAGATTAACCGACAGATTCAATCTCGTGGATGGACGTTCAACATTGAGGAAGGCATAACGCTACTACCTGATGTTTACTCCAACCTGATTGTATACAGTGACGACTATTTATCCCTAATGTCTACTTCCGGTCAATCCATCTACGTTAACCGAGGTGGCTATGTG  >  NC_001604.1/23938‑24536`

  

Alignment Legend

Aligned base mismatch/match (shaded by quality score): `ATCG/ATCG` `< 3 ≤` `ATCG/ATCG` `< 9 ≤` `ATCG/ATCG` `< 20 ≤` `ATCG/ATCG` `< 34 ≤` `ATCG/ATCG` `< 38 ≤` `ATCG/ATCG`

Unaligned base: `atcg`    Masked matching base: `atcg`    Alignment gap: `‑`    Deleted base: `‑`
